# Effect of emergency major abdominal surgery on CD4 cell count among HIV positive patients in a sub Saharan Africa tertiary hospital - a prospective study

**DOI:** 10.1186/1471-2482-13-4

**Published:** 2013-02-26

**Authors:** Gabriel Okumu, Patson Makobore, Sam Kaggwa, Andrew Kambugu, Moses Galukande

**Affiliations:** 1 Kisizi Hospital, Kampala, Uganda; 2 Department of Surgery, College of Health Sciences, Makerere University, Kampala, Uganda; 3 Infectious Disease Institute, College of Health Sciences Makerere, Kampala, Uganda

**Keywords:** Major surgery, CD4, HIV

## Abstract

**Background:**

Surgery plays a key role in HIV palliative care, specifically in the diagnosis and treatment of HIV related and non-related conditions. Yet major surgery depresses the immune system. Whereas the surgical consequences of HIV infection are well described, there is a paucity of published data, in resource-limited settings, on the effects of major surgery on the immune system. The purpose of this study was to determine the effect of major abdominal surgery on CD4 count in HIV positive and HIV negative patients after emergency major surgery.

**Methods:**

A prospective cohort study was done for patients who underwent emergency major abdominal surgery. Their peri-operative CD4 counts were done for both HIV- and HIV + patients. Median CD4s were used in analysis.

Mann Whitney test of significance was used for continuous data and Fisher’ exact test used for categorical data. IRB approval was obtained.

**Results:**

A total of 101 patients were recruited, 25 HIV positive and 76 HIV negative. The median CD4 cell reduction was higher in the HIV negative group (−68 cells) than HIV positive group (−29 cells) (p = 0.480).

There was a general increase in the median CD4 change by 72 cells for the HIV positives and 95 cells for the HIV negatives (p = 0.44). CD4 change rose in both the HIV positive and negative groups by 27 cells for the HIV positives and 28 cells for the HIV negatives (p = 0.94). Relative Risk was 0.96, {CI 0.60 – 1.53}.

**Conclusion:**

Major emergency abdominal surgery had no significant effect on CD4 cell count among HIV positive patients.

## Background

Over 40 million people worldwide are believed to be living with HIV or AIDS [1]. HIV/AIDS remains a global problem despite all the effort and vast amount of money spent on controlling it in the last two decades.

HIV is a Lentivirus, a subgroup of retroviruses. This family of viruses is known for latency, persistent viremia, infection of the nervous system, and weak host immune responses. HIV has high affinity for CD4 T lymphocytes and monocytes. HIV binds to CD4 cells and becomes internalized. The virus replicates itself by generating a DNA copy by reverse transcriptase. Viral DNA becomes incorporated into the host DNA, enabling further replication [2].

Surgical intervention has become a common component in the management of patients infected with HIV or from clinical consequences of AIDS [2].

The advent of HAART in recent years has transformed HIV from a disease leading to rapid decline and early death into a chronic, manageable condition [3]. Consequently many HIV infected patients undergo surgeries for conditions that would not have been treated previously.

The earliest experiences regarding surgical procedures in AIDS patients reported complication rates as high as 40% and mortality rates ranging from 55% to 70% [4].

This information however has mainly been derived from previous retrospective studies and has mainly been carried out in the western world where the presentation of HIV is not exactly the same as that in sub-Saharan Africa where it’s estimated that about 60–70% of the world’s HIV infected patients are found [1].

Surgical decision making is complex when the patients is HIV positive and many surgeons recommend medical management or conservative approaches for conditions usually surgically treated [3,5].

The absolute number of circulating CD4 lymphocyte has been shown to be a clinically useful indicator of immune function in HIV infected individuals [6]. Because CD4 lymphocyte is a specific target of HIV, it serves as a marker for immune status and can be used as a predictor of lowered immunity.

The purpose of this study was to determine the impact of emergency major surgery on the absolute CD4 count among HIV positive compared to HIV negative patients at a sub Saharan African Hospital.

## Methods

### Study design

The study was a Prospective double Cohort study.

### Study setting

The study was conducted at Mulago national referral and teaching hospital, which is a teaching hospital for Makerere university medical school and other paramedical schools around it. It has a 1500 bed capacity and offers curative and preventive as well as rehabilitative care to patients. Patients were recruited from the emergency surgical ward.

### Study population

All patients due for emergency major surgical operations.

### Inclusion criteria

Patients with conditions requiring emergency major operation.

### Exclusion criteria

Patients having repeat major operations within one month of admission, patients with cancer or other immunosuppressive ailment such as diabetes mellitus, patients on cytotoxic drugs, and patients of ASA III and above.

Children less than18 months as all HIV testing was by antibody tests.

### Sampling

Consecutive sampling was used for both HIV positive and negative patients who met the inclusion criteria. Patients for emergency major surgery were reviewed in the emergency surgical ward and written informed consent obtained for the study, for HIV test and for the operation.

### Study variables

Major abdominal surgery and CD4 cell count.

### Sample handling

Blood for HIV test, CD4 count and CBC was drawn in sequestrin bottles containing EDTA under sterile conditions about 2 mls of blood were drawn. The samples were shaken gently to mix with the EDTA. Blood for CD4 count was taken between 7 pm to 11 pm because of the diurnal variation [4]. Consent for blood drawing and storage was obtained from patients.

The samples were labeled with; serial number, age, sex, in patient number and ward.

The samples were transported standing upright in a rack.

The samples collected in sequestrin bottles for HIV test and were kept at room temperature. Some stayed for up to 12 hours.

Samples for CBC were kept in the refrigerator for less than 24 hours. Storage of all samples was at the laboratory in Mulago hospital. Samples for CD4 were collected in sequestrin bottles and taken immediately to the laboratory. Samples taken late when the lab was closed were stored at room temperature for a maximum of 12 hours then taken to the laboratory the following morning. These samples can be kept safely at room temperature for a maximum of 18 hours [4].

Patients got their results after the surgery as the tests were carried out the following day. The HIV counselor gave the results to the patients after counseling. HIV positive patients with CD4 cell counts less than 200 cells/ml were referred to the nearest HIV palliative care clinic for care after discharge. Patients who accepted to enroll in the study but refused to receive their results were left in the study but not forced to get the results.

The laboratory test was done by one qualified laboratory technician in an accredited laboratory. There was a trained HIV counselor for all the patients.

### Data collection

Data was collected using standardized pre-tested and pre-coded interviewer administered questionnaires.

Samples were drawn at admission, a day after surgery and on the 7^th^ POD.

### Data analysis

Questionnaires were checked for completeness and accuracy. Data was entered using Epi Data statistical package and analyzed using STATA 10 statistical package.

Baseline characteristics were compared for the two groups. Diagnoses and operations for both groups were also compared and CD4 changes in the groups after different operations were studied.

After calculating the median pre-operative CD4 and median 1^st^ post-operative day CD4 then median 7^th^ post-operative CD4 counts, a non-parametric test was used for testing the hypothesis since the data wasn’t normally distributed.

A Mann Whitney test was used to test for statistical significance between changes in CD4 reduction e.g. between pre-operative value and 1^st^ post-operative day value and between any of these and the 7^th^ post-operative day value. Fisher’s exact test was used for small quantity categorical data.

Tables and figures were used to show the distribution of factors in the two groups.

Relative risk and adjusted Odds ratio were calculated.

### Ethical considerations

Informed written consent was obtained from the patients for the study and tests. Those under 18 years were consented by their parent or guardian.

Approval for the study was obtained from the school of medicine of Makerere University, the Mulago hospital ethics & research committee and the Uganda National Council of Science and Technology.

## Results

A total of 115 patients were recruited over a 5 months period had emergency major surgery in Mulago hospital emergency theatre.

Of the 110 patients who were finally enrolled in the study 82 were HIV negative and 28 were HIV positive. Of the 82 HIV negative patients 6 were lost to follow up, leaving 76 patients and of the 28 HIV positive patients 3 were lost to follow up leaving 25 whose data was included in the analysis (Table 1).

**Table 1:** Population demographic characteristics between HIV positive and negative patients groups, CD4 count study, 2012

**Characteristics**		**Frequency**		**p-value**
		**HIV positive (n = 25)**	**HIV negative (n = 76)**	
**Age**	<20	1 (4%)	14 (18.4%)	
	20–40	19 (76%)	40 (52.6%)	0.084
	>40	5 (20%)	22 (29%)	
**Gender**	Male	18 (72%)	54 (71.1%)	
	Female	7 (28%)	22 (27.9%)	0.941
**Marital status**	Married	13 (52%)	38 (50%)	
	Single	8 (32%)	36 (47.4%)	0.035
	Divorced/widowed	4 (16%)	2 (2.6%)	
**Occupation**	Peasant/farmer	8 (32%)	27 (35.5%)	0.079
	Self employed	5 (20%)	10 (13.2%)	
	Boda boda	7 (28%)	9 (11.8%)	
	Student/pupil	0	14 (18.4%)	
	Others	5 (20%)	16 (21.1%)	0.113
**Laboratory tests**				
Hemoglobin (g/dl)	12.2 iqr (3.7)		13.4 iqr (3.55)	0.521
Total white blood cells (cells/ml)	7600 iqr (5600)		8750 iqr (5950)	0.450
Neutrophils (%)	68.8 iqr (18.2)		68.95 iqr (18.45)	0.0478
Lymphocytes (%)	14.2 iqr (14.7)		22.35 iqr (16.8)	0.795
Other WBC’s	17 iqr (10)		8.7 iqr (5.6)	

[iqr = Inter quartile range].

### Comparison of demographic characteristics of patients in the study

There were 15 patients under 20 years, 59 patients between 20–40 years and 27 patients over 40 years.

Among the three age groups, the under 20’s had the lowest number of HIV infection. The group with the highest infection was the 20–40 age group. The over 40’s age group had more HIV infection than those less than 20 years. There were 72/101 (71.3%) male patients and 29/101 (28.7%) female patients. In the HIV positives, males were more i.e. 18/25 (72%) compared to 7/25 (28%) females. In both male and females the prevalence of HIV was similar i.e. about 25%.

51 of the patients in the study were married, 44 were single and 6 were divorced or separated. The divorced/separated group had the biggest number of HIV positives (66.7%).

### Clinical observations and laboratory findings

Median hemoglobin level among patients studied was also almost similar in both study groups 13.4 g/dl in the HIV negatives and 12.2 g/dl in the HIV positives p = 0.11. Median white blood cell count was higher the HIV negative group 8750 cells/ml compared with 7600cells/ml in the HIV positives p = 0.52.

Between the pre-operative to 1^st^ POD there was a general CD4 fall in both groups with the highest fall for the HIV negative patients who had small gut resection and anastomosis (−429), followed by patients who had laparotomy with appendectomy and pus drainage (−155). In the HIV positive group the highest fall was in patients who had repair of perforated gut (−213), followed by those who had small gut resection and anastomosis (−152.9). HIV positive patients who had laparotomy with appendectomy and pus drainage had a rise in their CD4 count by 64 cells.

Between the 1^st^ and 7^th^ POD there was a general rise in the CD4 cells. In the HIV negatives the highest rise was in the patients who had laparotomy with appendectomy and pus drainage with (+342 cells), and in the HIV positive group the highest rise as in patients of perforated PUD repair (+314.5 cells) (Table 2).

**Table 2:** Median CD4 changes in the different age groups

	**Median CD4 change in HIV-ve**				**Median CD4 change in HIV + ve**			
**Age groups**	**No**	**Pre – 1**^**st **^**POD**	**1**^**st **^**– 7**^**th **^**POD**	**−7**^**th **^**POD**	**No**	**Pre - 1**^**st **^**POD**	**1**^**st **^**– 7**^**th **^**POD**	**7**^**th **^**POD**
< 20	14	- 210	+ 79	- 86.5	1	+ 240	+ 37	+ 277
20–40	40	- 49.5	+ 77.5	+ 28	19	- 29	+ 65	+ 26
> 40	22	- 70.5	+ 208.5	+ 161.5	5	- 120	+ 186	+ 78
**Gender**								
Male	54	- 56	+ 95	+ 39	18	- 55	+ 77.5	+ 14.5
Female	22	- 101.5	91.5	- 20	7	- 6	+ 32	+ 78

iqr = interquartile range of median value.

### CD4 change with age

The CD4 changes with age were almost similar in both groups. Between the pre-operative days to 1^st^ POD the CD4 generally dropped except for the only HIV positive patient who was under 20 years of age (+240 cells). It’s also noted that the greatest drop was in the HIV negative patients less than 20 years (−210 cells).

Between 1^st^ and the 7^th^ POD’s all CD4 cells rose. Between pre-operative to 7th POD the only drop was in the HIV negative patients less than 20 years (−86.5). All the rest showed increase as shown in Table 3 below.

**Table 3:** Two by two table for calculating relative risk

	**HIV status**		**Total**
	**Exposed (HIV + ve)**	**Unexposed (HIV-ve)**	
	**N = 25**	**N = 76**	
Cases (CD4 fall > =64)	12	38	50
Non-cases (CD4 fall <64)	13	38	51
Total	25	76	101
Risk	0.48	0.5	0.50
		[95% Conf Interval]	
Risk difference	−0.02	- 0.25 - 0.21	
Relative risk	0.96	0.60 - 1.53	
Odds ratio	0.92	0.38 - 2.25	

The median CD4 fall in both HIV positive and negative groups was 64 cells/ml.

Fishers exact p-value = 1.00.

### CD4 change between males and females

Between pre-operative day to 1^st^ POD the female HIV negative patients had a greater reduction in CD4 count (−210 cells) while in the HIV positives, male patient’s had a greater CD4 reduction pre-operative to 1^st^ POD (−55 cells).

All other CD4 cells were increasing except for the females in the pre-operative to 7^th^ POD (−20 cells).

### Comparison of median CD4 count results pre-op, 1^st^ POD and 7^th^ POD

In general comparing CD4 count between HIV positive and HIV negative patients in the study, the CD4 counts were higher in the HIV negative patients as expected.

The median pre-operative CD4 counts in the HIV positive patients were above 200 cells/ml, the cut off below which HIV patients are considered to be at risk of developing AIDS, this signifies that the patients enrolled had good immunity and many hadn’t developed AIDS.

In the pre-operative median CD4 count the HIV negative were all above 50 cells with a good number in the 251–750 and >1000 ranges.

Median CD4 reduction was greatest between the pre-operative and 1^st^ POD in both HIV positive and negative patients. The reduction was however greater in HIV negative patients (−68 cells/ml). Medians values were used since the data wasn’t normally distributed.

Between the 1^st^ and 7^th^ post-operative days the CD4 levels increased more in HIV negative than in the positive patients.

**Figure 1:**
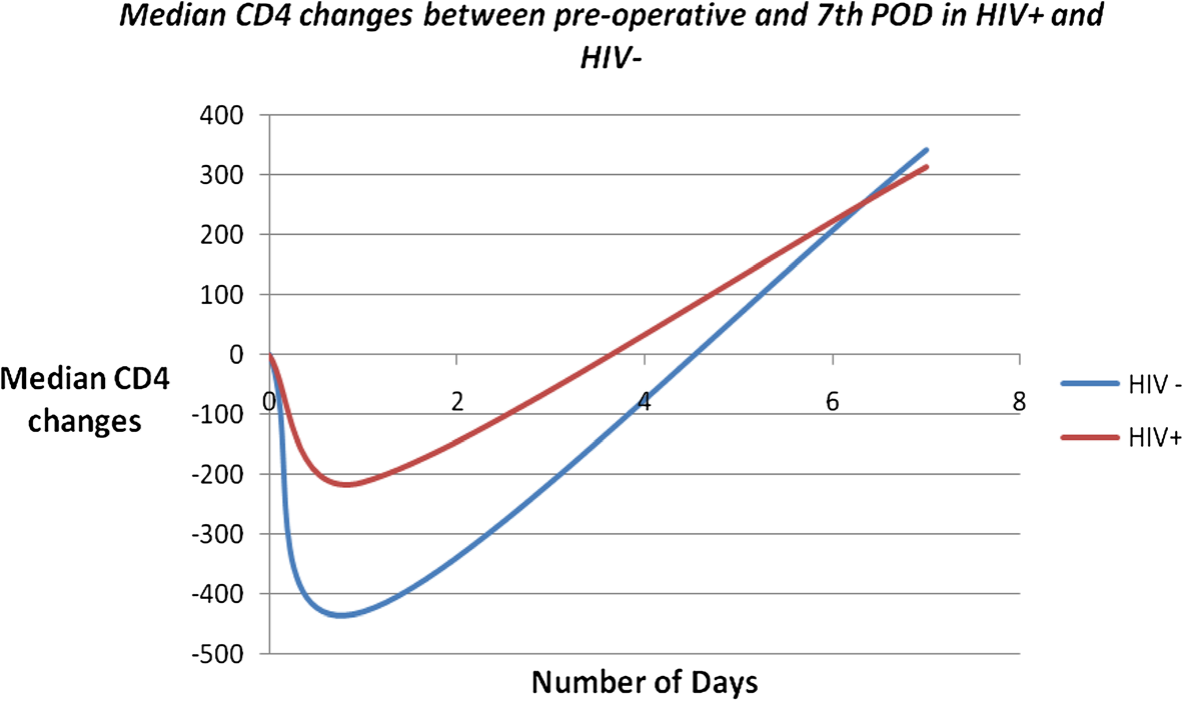
**Median CD4 changes from pre to 7**^**th **^**POD.**

There was also a general rise in CD4 levels between the preoperative level and the 7^th^ post-operative days which was almost the same in both groups. P-values were all way over 0.05 (Figure 1).

### Relative risk

Taking HIV status as the exposure and CD4 fall as cases the median CD4 reduction was 64 cells. Cases were those with CD4 fall greater or equal to 64 and non-cases were those with CD4 fall less than 64.

Relative risk was 0.96 which is less than 1, thus HIV exposure wasn’t a significant risk factor for CD4 reduction.

### Complications

Sepsis was the commonest complication in both study groups followed by anemia. Burst abdomen and anastomotic leak occurred in only the HIV negative group.

5 (20%) of the HIV positive patients got sepsis compared with 7 (9.2%) in the HIV negative group. 12% of the HIV positive patients developed anemia compared to 5.2% in the HIV negative group.

## Discussion

This study set out to investigate the influence of emergency major abdominal surgery on CD4 count among HIV positive patients. We found that major surgery did not significantly reduce CD4 count.

The exact mechanism of CD4 reduction is said to be due to several factors; cell lysis, autoimmune mechanism, anergy, effect of super antigens, apoptosis and virus specific immune responses [6].

Auto immune responses may be evoked by shared structural homology between MHC class II molecules and cellular humoral immune responses directed towards HIV proteins which cross react against self HLA antigens on T cells causing immune destruction.

The role of anergy in CD4 dysfunction is by binding of the glycoprotein 120 to CD4.

Molecules causing them to be refractory to further stimulation and destruction by HIV.

Super antigens are microbial or viral antigens capable of activation of many T-cells, in HIV infection; they render T-cells more susceptible to HIV.

All these factors are worsened by stress which is caused by surgical trauma. Other factors like infection and use of steroids can also trigger these factors.

The CD4 cell counts were generally lower in the HIV infected group than in the HIV uninfected group.

The high values of the CD4 count even in the HIV positive i.e. higher than 200 cells/ml the AIDS defining level of CD4 by WHO, can explain the finding that surgery had no statistically significant effect of reduction in CD4 as the pre-operative CD4 levels were high (347 cells/ml).

The blood for CD4 test was taken between 7 pm and 11 pm to cater for the diurnal variation. The CD4 reduction was transient in our study with CD4 levels rising to above pre-operative values on the 7^th^ POD in both the HIV positive and negative patients. This was similar to previous studies by Rahal [7], where it lasted 8 days, Bolla and Tuzzato [8] where it lasted 8 days and Johanna [9] where CD4 returned to baseline value between 6^th^ and 8^th^ post-operative days.

Surgery and anesthesia cause T-cell apoptosis which is said to be only transient lasting up to 5 days [10].

In our study emergency major surgery caused transient reduction in CD4 because within seven days the levels had risen to above pre-operative values. In a study by Ramon [11], CD4 drop persisted for only two days after which there was a rise. This was independent of the type of operation done. The reduction is said to be because stimulation and production of T-cells is reduced in the immediate post-operative period causing a CD4 drop. The rise in CD4 later occurs when stimulation & production increase.

Women showed a greater CD4 cell reduction than men in our study unlike in a study by Wichmann where men suffered longer lasting depression of CD4 than women of about 5 days while the depression in the women the CD4 depression lasted only 2 days [12].

In our study, the men had a better rise in CD4 count on 7^th^ POD than the women who instead had a reduction. This could be due to the fact that women tend to have other factors affecting their CD4 changes, like the menstrual cycle alone is said to have an effect on women’s CD4 count variations, [13]. Use of oral contraception’s also affects the CD4 count changes in women. Probably a combination of these factors could have caused these changes.

The CD4 reductions after surgery were highest in the HIV negatives than positives on 1^st^ POD which was unexpected as the HIV infection itself reduces the CD4 count. This could be explained by the different operations in the two groups as the greatest drop in CD4 count was different for different operations. It could also be due to the many factors causing CD4 variation. CD4 levels are said to vary by about 25% even in HIV negative patients [4].

Available information suggests that HIV positive patients should have a higher reduction in CD4 count because of the effect of the disease itself in addition to the other factors affecting CD4 count [6].

CD4 rise was more in the HIV negative patients in this study between 1^st^ and the 7^th^ POD and pre-operative to 7^th^ POD as expected (p > 0.05). In comparison, a study by Rahal [7] also showed that surgery does not affect immune function adversely in HIV-infected patients, as judged by CD4 cell counts or viral titers which were also done in their study.

In the Rahal study, of the 17 patients with CD4 cell counts >500/mm3 prior to surgery, 64.7% had unchanged counts after surgery (95% confidence interval [CI] 32.9%, 81.6%), whereas 35.2% of patients had lower CD4 counts after surgery (95% CI 14.2%, 61.7%).

Most patients in this study were in the (20–40) age group and most of HIV positive patients were in the same age group. This is expected as this is the most sexually active of the three categories of age in the study <20, 20–40 and >40. The general age range of the patients in the study was 10 years to 82 years with a median age of 35 years.

There were more males than females in the study and most of the patients in the study were married. The commonest operations in the study were appendectomy then sigmoidectomy for both groups which is almost similar to the study by Rahal [7] were the commonest operation was also appendectomy followed by hernia repair for that study. Other studies vary in scope of procedures done [14,15].

It would be expected that the reduction in the HIV positives be greater. This may be due to hyper stimulation of immune cells in the presence of infection in the HIV positives.

But in the same group the HIV negative show a better rise in CD4 between 1^st^ to the 7^th^ POD and a much greater rise than the HIV positives between pre-operative period to the 7^th^ post-operative day. This shows that their immune system recovered better than that of the HIV positive patients which is an expected finding.

Prevailing literature on effects of splenectomy on CD4 count suggests that splenectomy causes an abrupt and prolonged increase in CD4 cell count [4]. However in our study the 3 patients who had splenectomy who were all HIV negative had a marked fall in CD4 count by 1^st^ POD (Table 3), with a small rise between 1^st^ to 7^th^ POD and a fall between pre-operative to 7^th^ POD. This may be due to other stresses as all these patients were trauma patients with multiple injuries. It could be also due to the fact that CD4% as opposed to CD4 cell count is more accurate in assessment of asplenic patients [4].

### Limitations

This study wasn’t without limitations. There were several confounders not controlled for the clinical staging of HIV was not done, the period of starvation was not considered. Perhaps some HIV negatives were in the window period and should have been in the positives group. The indications of surgery were taken together; owing to small numbers meaningful stratification wasn’t possible. These limitations could have been controlled for to some extent by the selection process (which was random) and the comparison of the two groups showed comparability (insignificant p-values) for some important variables such as age, occupation and haemoglobin. The state of receiving HAART or being HAART naive was not considered. Over all we believe that our findings support the policy that HIV positivity alone should not deter or defer surgery when it clearly indicated, similar to cacala’s findings [14].

## Conclusion

Emergency major abdominal surgery had no significant effect on CD4 count in HIV positive patients. Emergency surgery should not be withheld or delayed when indicated among HIV positive patients.

## Abbreviations

CD4: Cluster of differentiation 4; HIV: Human immunodeficiency virus; AIDS: Acquired immune deficiency syndrome; HAART: Highly active anti-retroviral treatment; POD: Postoperative day; CI: Confidence interval; CBC: Complete blood count; DNA: Deoxyribonucleic acid; EDTA: Ethylenediaminetetraacetic acid; PUD: Peptic ulcer disease

## Competing interests

The authors declare that they have no competing interests.

## Authors’ contributions

OG originated the concept and collected data MP prepared the first draft OG, GM, KS and KA contributed to revising drafts and critical review of intellectual content. All Authors approved the final manuscript.

## Pre-publication history

The pre-publication history for this paper can be accessed here:

http://www.biomedcentral.com/1471-2482/13/4/prepub
